# Supplementary material for: Individual Neuronal Subtypes Exhibit Diversity in CNS Myelination Mediated by Synaptic Vesicle Release
Source: Curr Biol. 2016 Jun 6;26(11):1447–55. doi: 10.1016/j.cub.2016.03.070 (PMC4906267; doi:10.1016/j.cub.2016.03.070)
Supplement: Document S1. Supplemental Experimental Procedures, Figures S1–S3, and Table S1 [file mmc1.pdf]

Current Biology, Volume *26*

Supplemental Information

Individual Neuronal Subtypes Exhibit Diversity in CNS Myelination Mediated by Synaptic Vesicle Release

Sigrid Koudelka, Matthew G. Voas, Rafael G. Almeida, Marion Baraban, Jan Soetaert, Martin P. Meyer, William S. Talbot, and David A. Lyons

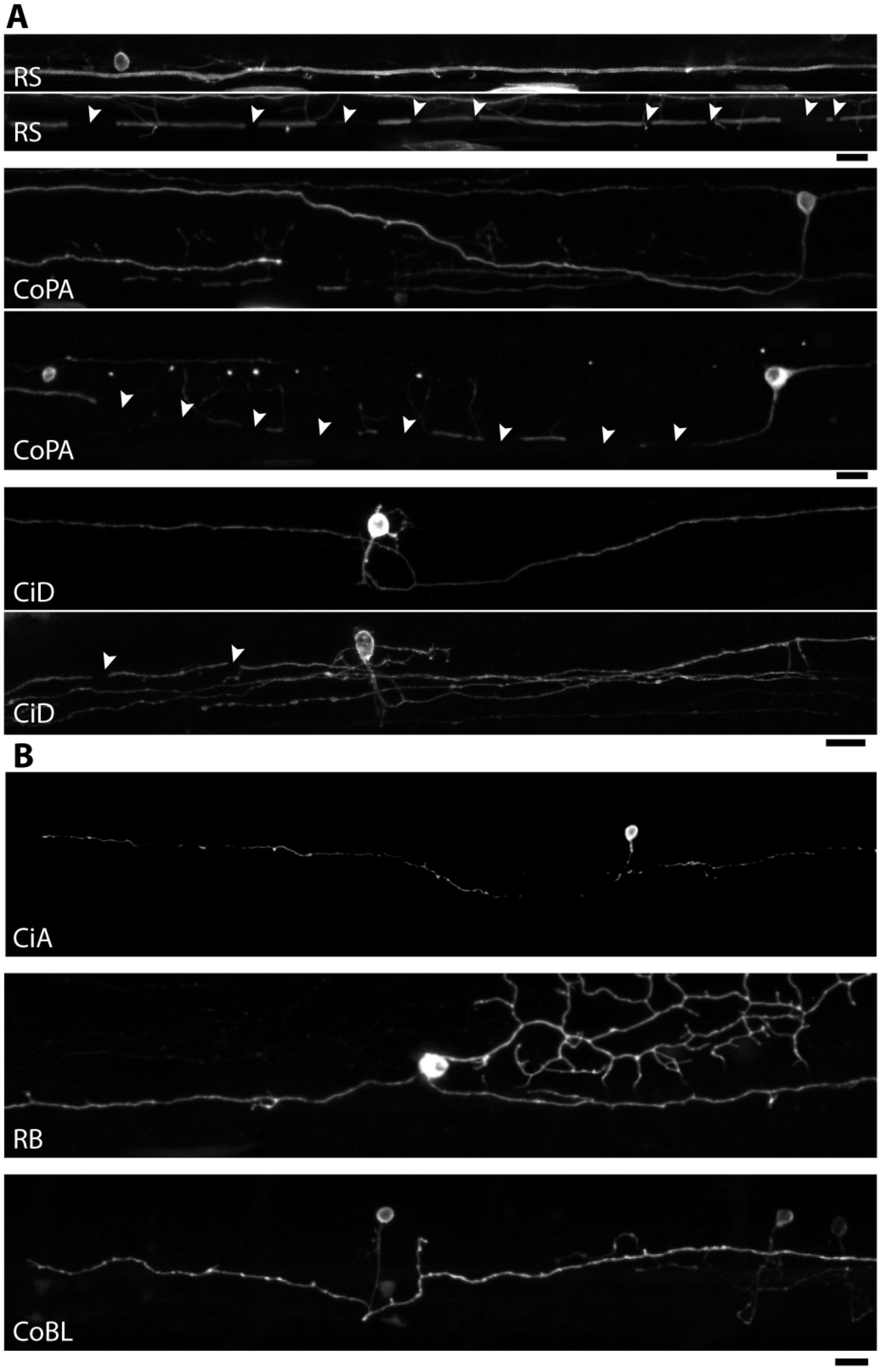

Figure S1 (relates to Figures 1, 2, 3).

Overview of individual subtypes labelled with GFP-Cntn1a that have myelinated axons (A) and that do not have myelinated axons (B) over the stages examined. A shows an example of each neuronal subtype at stages prior to myelination and upon myelination. Arrows point to position of myelin sheaths.

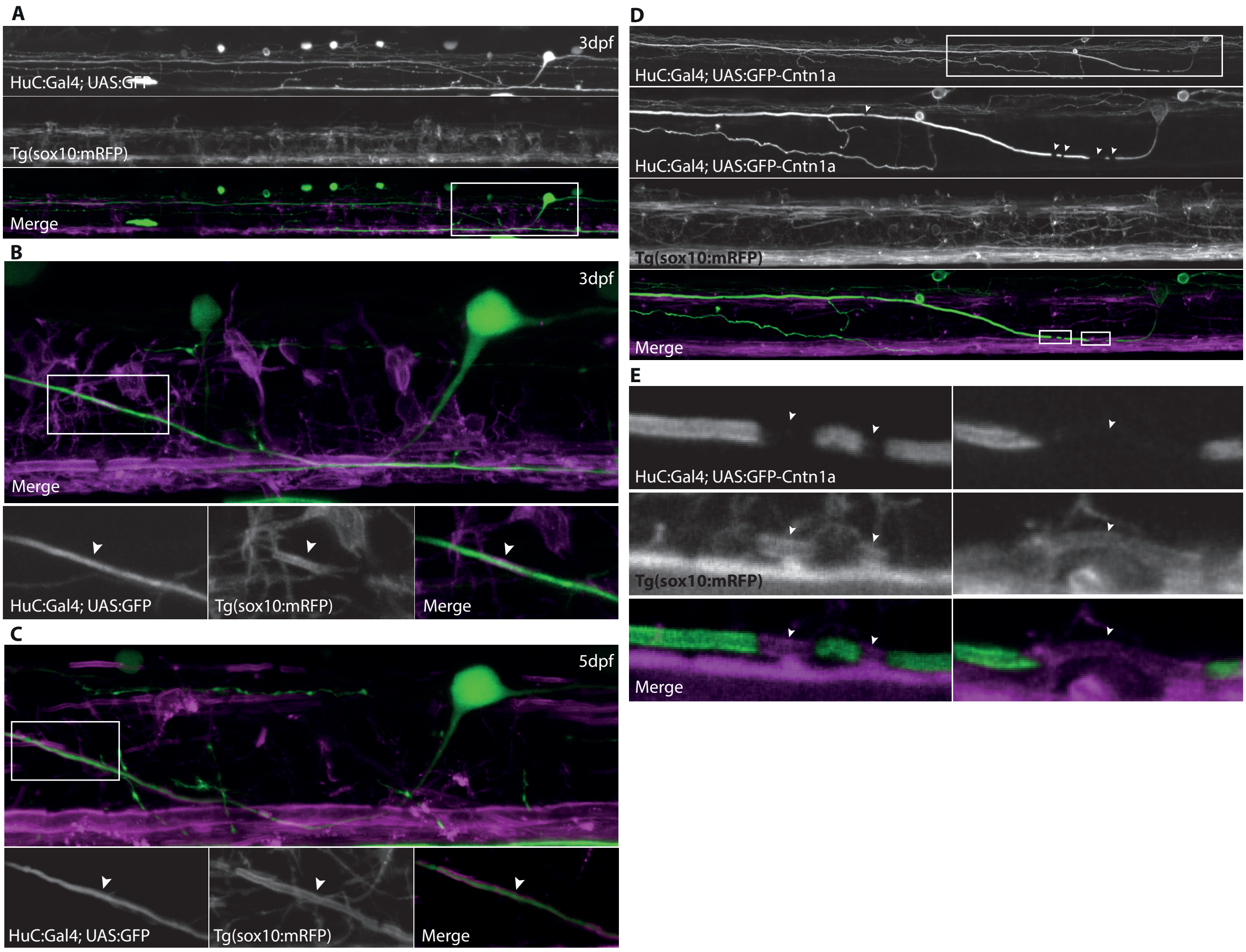

Figure S2 (relates to Figures 1 and 3).

Left panels: Individual CoPA neuron lablelled with cytoplasmic GFP in a Tg(sox10:mRFP) animal, imaged with a 10x magnification objective lens at 3dpf (A) and 63x (B) and also at 5dpf (C). Arrows indicate myelin sheaths. Area imaged in B outlined by inset in A.

Right panels: Individual CoPA neuron lablelled with GFP-Cntn1A in a Tg(sox10:mRFP) animal, imaged with a 10x magnification objective lens (D, top panel) 20x (D panels 2-4) and 63x (E). Arrows indicate myelin sheaths. Areas imaged in D outlined by insets in E.

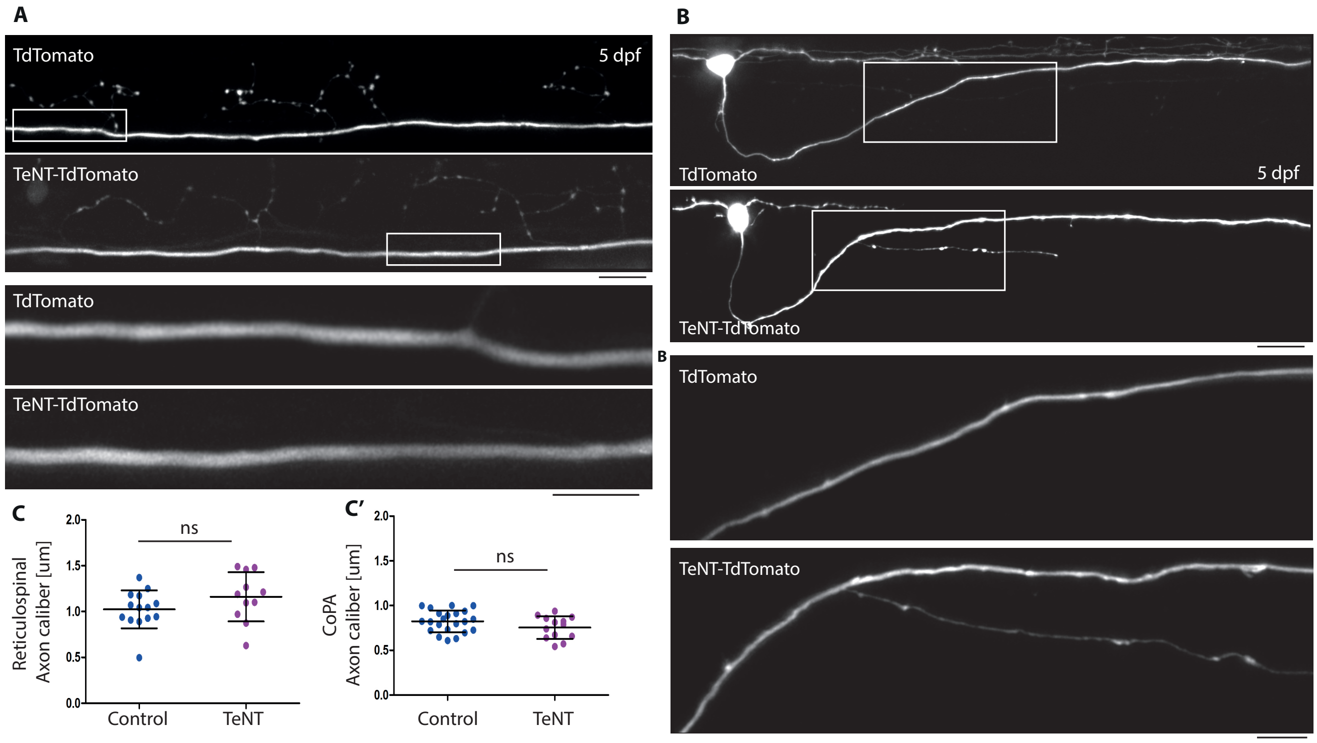

Figure S3 (relates to Figures 2, 3)

Individual reticulospinal axons (A) are of similar caliber when expressing control TdTomato (top) alone or TeNT-TdTomato (second from top) at 5 dpf. Scale bar: 15µm. Areas outlined by boxes in top and second panels are shown imaged by Airyscan in 3rd and 4th panels respectively. Scale bar: 5µm.

Individual CoPA axons (B) are of similar caliber when expressing control TdTomato (top) alone or TeNT-TdTomato (second from top) at 5 dpf. Scale bar: 20µm. Areas outlined by boxes in top and second panels are shown imaged by Airyscan in 3rd and 4th panels respectively. Scale bar: 5µm.

C and C' show quantification of axonal caliber and reveal no difference between control and TeNT expressing reticulospinal neurons (C) or CoPA (C').

Neuron cell type	Picture of neuron	Soma postition / # per hemi-segment	Maximum axon length	% of neurons with myelinated axons
Reticulospinal	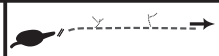	Ventral midbrain & hindbrain / axon projects through length of the spinal cord	Extends length of cord.	Myelinated: 72% (28 out of 39)
Commisural Primary Ascending (CoPA)	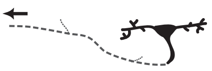	Dorsal, lateral to CoLA cell bodies / cell # varies in alternate segments	Extends length of cord / axon crosses cord dorsal to Mauthner axon. Ascend in dorsal cord.	Myelinated: 82% (35 out of 43)
Circumferental Descending (CiD)	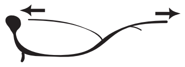	Midcord lateral / mutlitple cells per hemi-segment	13 segments/ axon ipsilateral, extends ventral to Mauthner axon then turns dorsal and caudally. Minor ascending branch.	Myelinated: 53% (38 out of 72)
Circumferental Ascending (CiA)	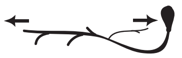	Midcord lateral / mutlitple cells per hemi-segment	Varies, the caudally projecting axon extends ipsilaterally over several segments. Minor descending branch.	Not myelinated: (0 out of 68)
Commisural Bifurcating Longitudinal (CoBL)	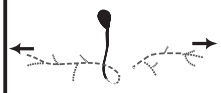	Dorsal / mutlitple cells per hemi-segment	4 segments/ axon projects ventrally, crossing cord dorsal to Mauthner axon before bifurcating and extending dorsally.	Not myelinated: (0 out of 40)
Rohon Beard (RB)	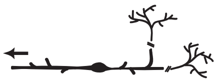	Dorsal of all other cells / 1-4 cells per hemi-segment	Caudally projecting axon extends > 20segments. Rostrally projecting axon extends 10 segments.	Normally not myelinated: 4% (2 out of 48, one short sheath each)

Table S1 (relates to Figures 1, 2, 3).

Summary of single neuron myelination profiling.

Table outlines neuronal subtypes whose myelination status at embryonic-early larval stages (up to 7 days post-fertilisation) was analysed, and provides schematic overview of characteristic morphology. The dashed lines indicate axons that cross the embryonic midline and double forward slashes indicate long distances between areas shown. Arrows indicate direction of axonal projection with anterior to the left and dorsal to the top.

Supplemental Experimental Procedures

In order to profile which neurons have myelinated axons during the first week of larval zebrafish development we mosaically labelled individual neurons with the GFP-Cntn1A transgenic reporter (Figure S1). We found that reticulospinal neurons, CoPA neurons and CiD (Circumferential Descending) neurons had myelinated axons during the first week of larval life (Figure S1+Table S1). To validate our results we also examined the myelination of individual neurons labelled by cytoplasmic GFP in a Tg(sox10:mRFP) background (Figure S2). Both analyses revealed prominent myelination of reticulospinal, CoPA and CiD neurons in the first week of life and no myelination of Circumferential Ascending, Commissural Bifurcating Longitudinal, and Rohon Beard axons (Table S1). Although these neurons did not have myelinated axons at the stages examined, we cannot rule out a later onset of myelination in these neurons. Furthermore, our analyses did not profile the myelination of every neuronal subtype with axons in the embryonic/ larval spinal cord, which will be important in future studies. Super-resolution imaging analyses of axonal caliber showed that control (TdTomato) and vesicular release impaired (TeNT-TdTomato) expressing neurons were of similar size (Figure S3).
