# Supplementary figures and images for: Individual Neuronal Subtypes Exhibit Diversity in CNS Myelination Mediated by Synaptic Vesicle Release
Source: Curr Biol. 2016 Jun 6;26(11):1447–55. doi: 10.1016/j.cub.2016.03.070 (PMC4906267; doi:10.1016/j.cub.2016.03.070)

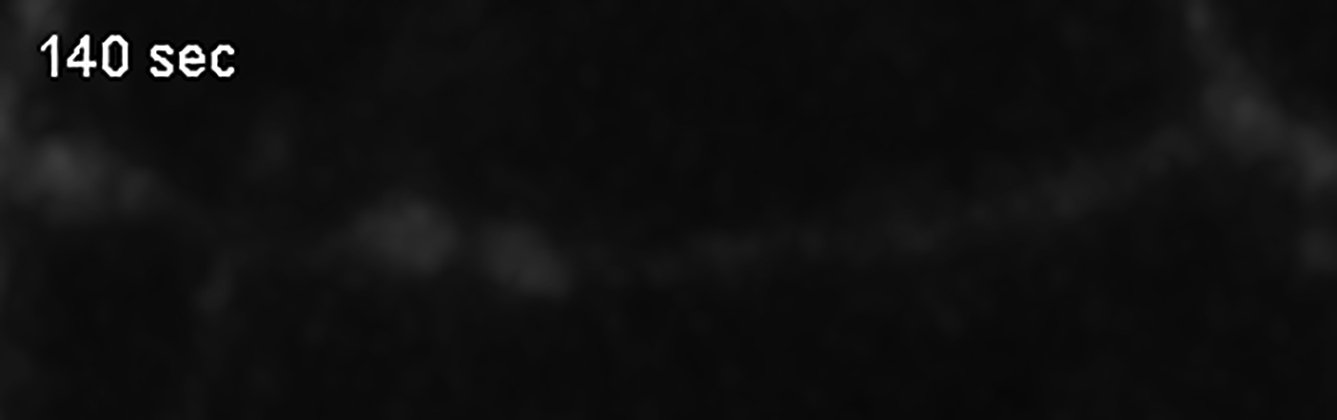

Supplement: Movie S1. Time-Lapse Analyses of sypHy Expressing Control Reticulospinal Neuron Axonal Collateral, Related to Figure 4 — Arrowheads indicate increases in pHluorin intensity, indicating vesicular release events. Times of bleaching events and frame duration noted. [file mmc2.jpg]

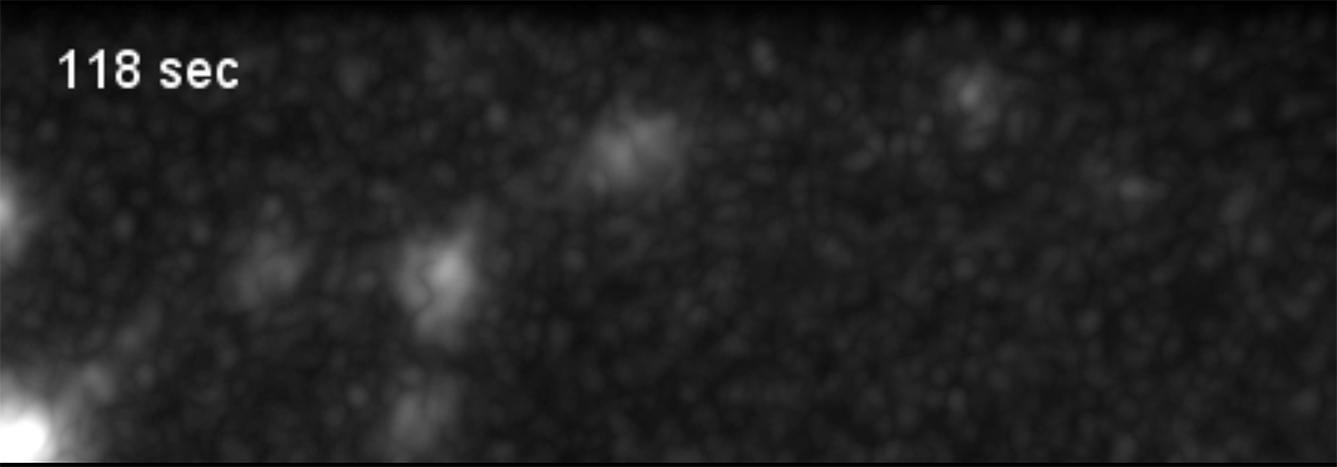

Supplement: Movie S2. Time-Lapse Analyses of sypHy Expressing a TeNT Expressing Reticulospinal Neuron Axonal Collateral, Related to Figure 4 — Times of bleaching events and frame duration noted. [file mmc3.jpg]

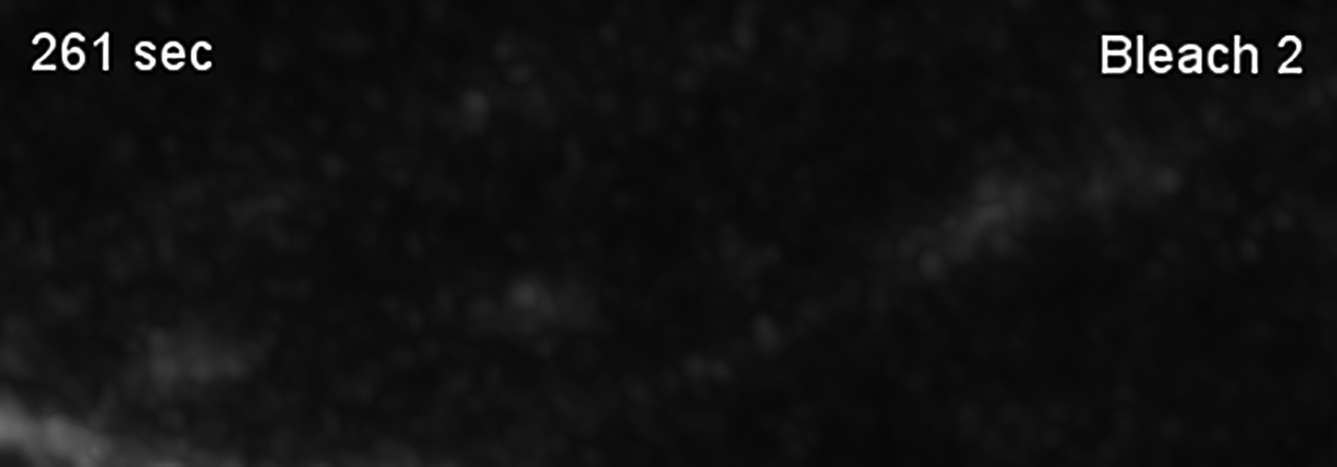

Supplement: Movie S3. Time-Lapse Analyses of sypHy Expressing Control CoPA Neuron Axonal Collateral, Related to Figure 4 — Arrowheads indicate increases in pHluorin intensity, indicating vesicular release events. Times of bleaching events and frame duration noted. [file mmc4.jpg]

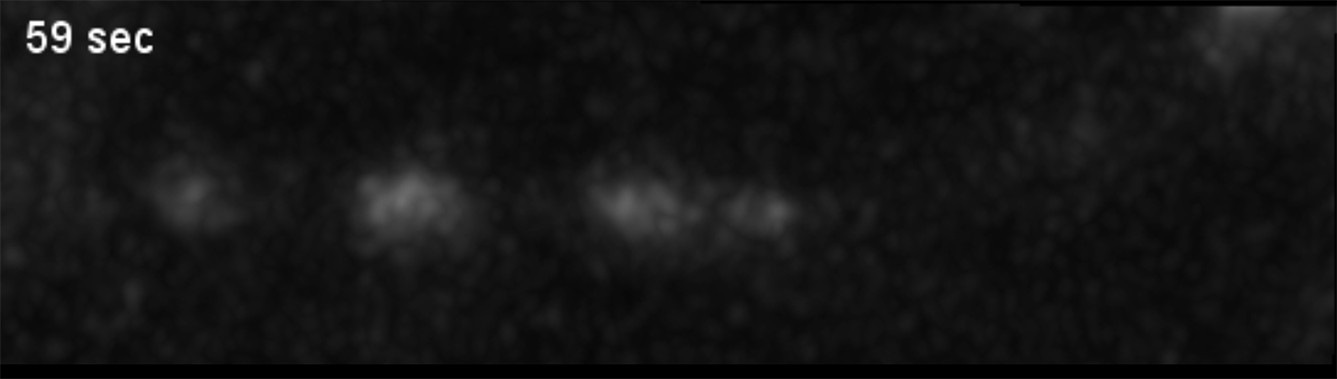

Supplement: Movie S4. Time-Lapse Analyses of sypHy Expressing TeNT Expressing CoPA Neuron Axonal Collateral, Related to Figure 4 — Times of bleaching events and frame duration noted. [file mmc5.jpg]
