# Supplementary material for: Individual Neuronal Subtypes Exhibit Diversity in CNS Myelination Mediated by Synaptic Vesicle Release
Source: Curr Biol. 2016 Jun 6;26(11):1447–55. doi: 10.1016/j.cub.2016.03.070 (PMC4906267; doi:10.1016/j.cub.2016.03.070)
Supplement: Document S2. Article plus Supplemental Information [file mmc6.pdf]

Report

Current Biology

Individual Neuronal Subtypes Exhibit Diversity in CNS Myelination Mediated by Synaptic Vesicle Release

Highlights

- Live imaging of myelin sheath dynamics along single axons over time in zebrafish
- Vesicular release from reticulospinal axons required for normal myelination
- Vesicular release from CoPA axons not required for myelination
- Evidence of neuronal subtype diversity of activity-regulated myelination

Authors

Sigrid Koudelka, Matthew G. Voas, Rafael G. Almeida, ..., Martin P. Meyer, William S. Talbot, David A. Lyons

Correspondence

david.lyons@ed.ac.uk

In Brief

Neuronal activity can regulate myelination, which in turn affects nervous system function. Koudelka et al. use zebrafish to image myelination over time along individual axons in vivo. They find that while some neuronal subtypes regulate myelination by vesicular release, others do not, indicating striking diversity in activity-regulated myelination.

Koudelka et al., 2016, Current Biology *26*, 1447–1455
June 6, 2016 © 2016 The Authors. Published by Elsevier Ltd.
http://dx.doi.org/10.1016/j.cub.2016.03.070

Current Biology
Report

CellPress

Individual Neuronal Subtypes Exhibit Diversity in CNS Myelination Mediated by Synaptic Vesicle Release

Sigrid Koudelka,[1] Matthew G. Voas,[2] Rafael G. Almeida,[1] Marion Baraban,[1] Jan Soetaert,[1] Martin P. Meyer,[3] William S. Talbot,[2] and David A. Lyons[1,*]
[1]Centre for Neuroregeneration, MS Society Centre for Translational Research, Euan MacDonald Centre for Motor Neurone Disease Research, University of Edinburgh, Edinburgh EH16 4SB, UK
[2]Department of Developmental Biology, Stanford University, Stanford, CA 94305, USA
[3]MRC Centre for Developmental Neurobiology, New Hunt's House, King's College London, Guy's Hospital Campus, London SE1 1UL, UK
*Correspondence: david.lyons@ed.ac.uk
http://dx.doi.org/10.1016/j.cub.2016.03.070

SUMMARY

Regulation of myelination by oligodendrocytes in the CNS has important consequences for higher-order nervous system function (e.g., [1–4]), and there is growing consensus that neuronal activity regulates CNS myelination (e.g., [5–9]) through local axon-oligodendrocyte synaptic-vesicle-release-mediated signaling [10–12]. Recent analyses have indicated that myelination along axons of distinct neuronal subtypes can differ [13, 14], but it is not known whether regulation of myelination by activity is common to all neuronal subtypes or only some. This limits insight into how specific neurons regulate their own conduction. Here, we use a novel fluorescent fusion protein reporter to study myelination along the axons of distinct neuronal subtypes over time in zebrafish. We find that the axons of reticulospinal and commissural primary ascending (CoPA) neurons are among the first myelinated in the zebrafish CNS. To investigate how activity regulates myelination by different neuronal subtypes, we express tetanus toxin (TeNT) in individual reticulospinal or CoPA neurons to prevent synaptic vesicle release. We find that the axons of individual tetanus toxin expressing reticulospinal neurons have fewer myelin sheaths than controls and that their myelin sheaths are 50% shorter than controls. In stark contrast, myelination along tetanus-toxin-expressing CoPA neuron axons is entirely normal. These results indicate that while some neuronal subtypes modulate myelination by synaptic vesicle release to a striking degree in vivo, others do not. These data have implications for our understanding of how different neurons regulate myelination and thus their own function within specific neuronal circuits.

RESULTS AND DISCUSSION

Transgenic Reporter to Visualize Myelination along the Length of Single Axons In Vivo

To be able to study the dynamic formation and regulation of myelination along single axons over time, we needed to develop a new transgenic reporter. We reasoned that if we could label neuronal proteins that are initially localized along the entire axolemma and excluded from myelinated regions upon myelination, we might be able to assess myelination along single axons over time in vivo. Therefore, we generated a fluorescent fusion protein that combined GFP with the glycosylphosphatidylinositol (GPI)-anchored axonal protein Contactin1, which is localized broadly along the length of axons prior to myelination and to the nodal-paranodal region concomitant with myelination [15]. We found that expression of the construct GFP-contactin 1A in neurons (see the Experimental Procedures) resulted in robust localization of GFP along the axolemma prior to myelination. Importantly, upon glial contact, as assessed by the red fluorescent reporter Tg(sox10:mRFP), we found that GFP-Contactin1A was excluded from the positions of sox10:mRFP localization and myelin sheath formation (Figures 1A and 1B). Quantitative analyses indicated that all gaps in GFP-Contactin1A localization along axons were filled by sox10:mRFP-expressing profiles (119/119 gaps along 16 axons, n = 15 animals). Indeed, we found cases in which only a partial ensheathment of an axon by a putative myelinating process excluded GFP-Contactin1A expression precisely from that area of the axolemma (Figure 1B). We also observed GFP-Contactin1A localized to very short gaps between adjacent myelin sheaths at putative nodes of Ranvier (Figure 1C) [16]. In addition, we found that gaps in GFP-Contactin1A along single axons were also filled by myelinating processes labeled by the stable transgenic reporter mbp:mCherry-CAAX (Figure 1D). It is important to note that analysis of GFP-Contactin1A alone does not allow assessment of the radial growth of myelin sheaths. In summary, we can use GFP-Contactin1A (hereafter GFP-Cntn1a) to infer the presence and length of myelin sheaths along the axons of individual neurons in the living zebrafish over time.

CrossMark

This is an open access article under the CC BY license (http://creativecommons.org/licenses/by/4.0/).

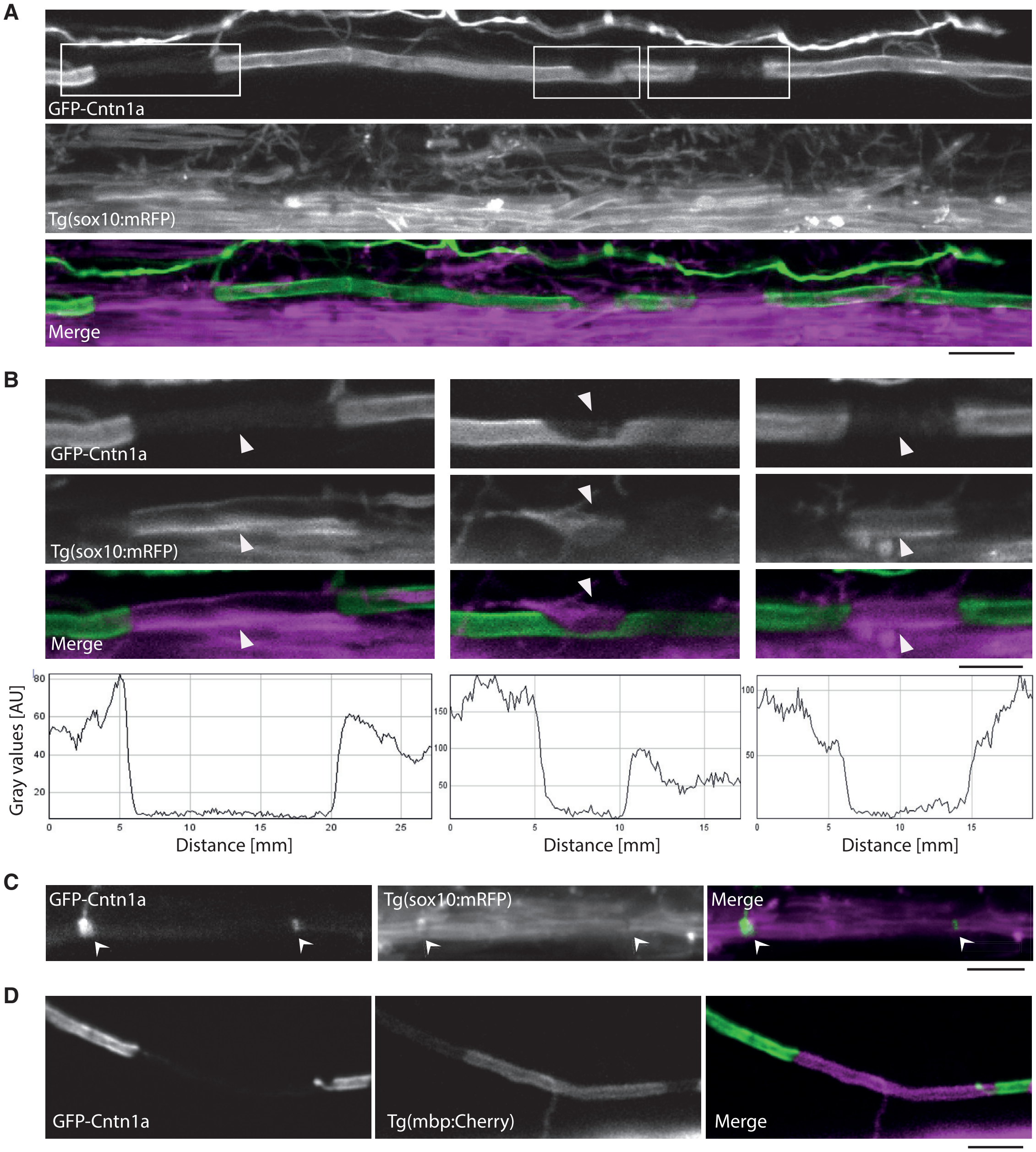

Figure 1. GFP-Cntn1a as a Tool to Visualize Myelin Sheaths along Single Axons

(A) Reticulospinal axon labeled with GFP-Cntn1a (top) in a Tg(sox10:mRFP) embryo, which labels oligodendrocytes and their myelin sheaths (middle). The myelin sheaths along the reticulospinal axon are localized to the gaps in GFP expression. Scale bar, 20 μm.

(B) High-magnification views of areas outlined in (A) (top). Scale bar, 5 μm. GFP-Cntn1a fluorescent intensity profiles of the insets from (A) (bottom).

(C) GFP-Cntn1a expression clustered at putative nodes of Ranvier (left) (arrows), as indicated by gaps in Tg(sox10:mRFP) expression (middle and right). Scale bar, 20 μm.

(D) GFP-Cntn1A along a CoPA axon in a Tg(mbp:mCherry-CAAX) embryo at 4 dpf shows expression of the myelin reporter in the gap of GFP-Cntn1A localization. Scale bar, 5 μm.

See also Figures S1 and S2 and Table S1.

Reticulospinal Neurons and CoPA Neurons Have Myelinated Axons in the Larval CNS

We exploited knowledge of distinct neuronal subtypes in the zebrafish CNS (e.g., [17, 18]) to profile myelination of their axons. Using a neuronal promoter (see the Experimental Procedures), we sparsely and randomly labeled neurons with the GFP-Cntn1a fusion protein, allowing us to visualize both neuronal morphology and myelination. Our in-depth studies of six distinct neuronal subtypes born early in the embryonic and larval CNS (Figures 2, 3, S1, and S2; Table S1) revealed that the first axons

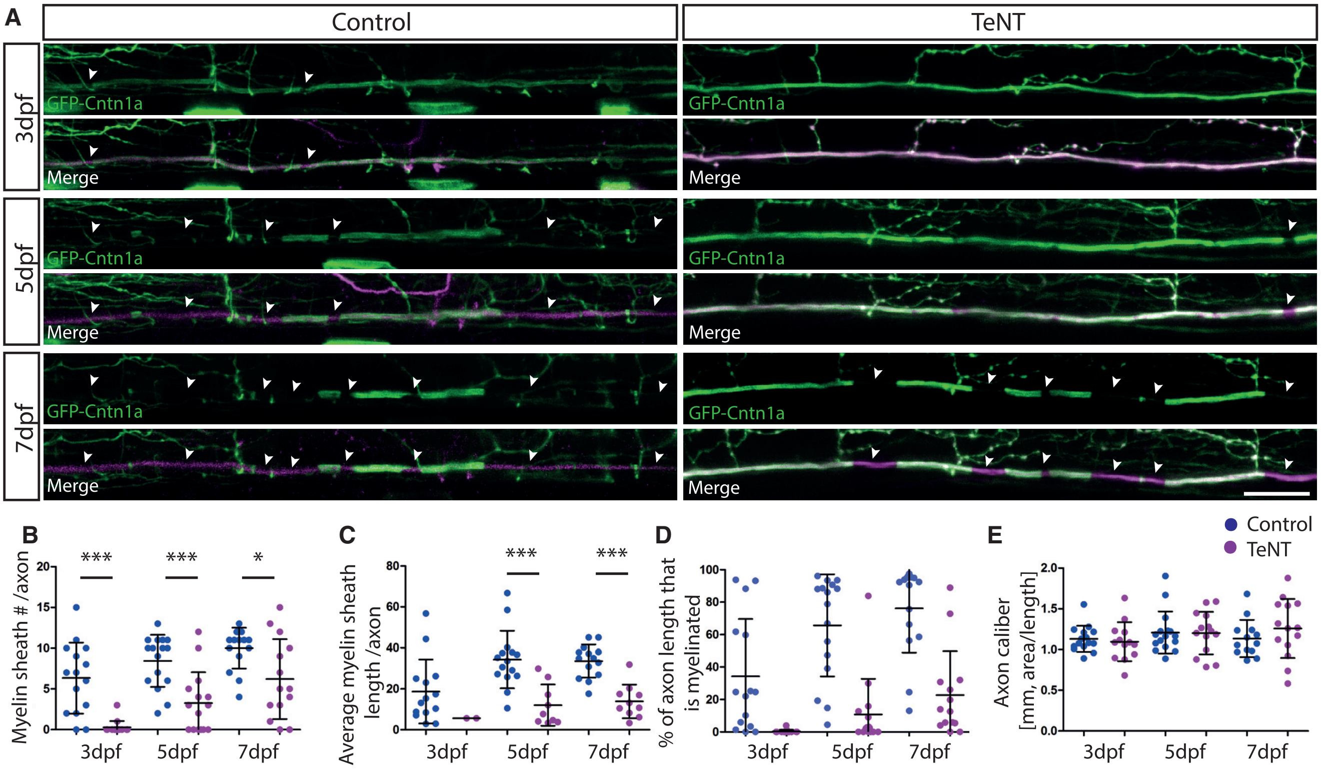

Figure 2. TeNT Expression in Reticulospinal Neurons Impairs Myelination along Individual Axons

(A) Individual reticulospinal axons labeled with GFP-Cntn1a and TdTomato (left) and with GFP-Cntn1a and TeNT-Tdtomato (right) at 3 dpf, 5 dpf, and 7 dpf. Scale bar, 15 μm.

(B–E) Quantification of myelin sheath number per axon per 425-μm imaging window (B), average length of myelin sheath per axon (C), percentage of axon length (per 425-μm imaging window) that is myelinated (D), and axon caliber (E) at 3 dpf, 5 dpf, and 7 dpf in control and TeNT-expressing reticulospinal neurons. All error bars indicate ± SD.

See also Figure S3.

myelinated included those of reticulospinal axons (Figures 2A and S1; Table S1), as previously indicated (e.g., [19]), and commissural primary ascending (CoPA) neurons (Figures 3A, S1, and S2; Table S1), interneurons that receive input from sensory neurons and project axons from the spinal cord to the brain [20, 21]. It is of note that both reticulospinal and CoPA neurons are born early [20, 22], are glutamatergic [23], and have axons of relatively large caliber (see below), as is characteristic for those that are myelinated first in the CNS in different species [19, 24]. Therefore, we decided to use reticulospinal and CoPA neurons to test whether different neuronal subtypes have similar or distinct modes of synaptic-vesicle-release-regulated myelination.

Vesicular Release Regulates Myelination along Reticulospinal Neurons

In order to test the role of vesicular release in regulating myelination over extended periods of time along individual reticulospinal axons, we expressed a tetanus toxin (TeNT)-TdTomato (TdT) fusion protein in single neurons. To visualize myelination along the length of single axons, we co-expressed the GFP-Cntn1a fusion protein with neurons expressing either TdTomato (TdT) or TeNT-TdTomato (TeNT-TdT) and assessed the myelination profile along single axons at three different time points (Figure 2). Our analyses revealed a striking reduction in myelination along the axons of reticulospinal neurons expressing TeNT-TdT compared to control TdT-expressing neurons. In a 425-μm-long imaging window, we found that at 3 days postfertilization (dpf), control reticulospinal axons had an average of 6.3 ± 4.3 myelin sheaths, with an average length of 19.9 ± 15.5 μm, covering 34% of the assessed axonal length (n = 15). In contrast, there were essentially no myelin sheaths on the axons of TeNT-expressing reticulospinal neurons at 3 dpf (n = 15, $p < 0.0001$) (Figures 2A and 2B). With our fluorescent reporters, we are able to follow individually labeled neurons and axons over time. At 5 dpf, the number and length of myelin sheaths were also greatly reduced by TeNT expression (sheath number: control 8.4 ± 3.2, n = 16 versus TeNT 3.3 ± 3.8, n = 15, $p = 0.0003$; average sheath length control 32.8 ± 14.9 μm versus TeNT 12.1 ± 10.1 μm, $p = 0.0012$) (Figures 2A–2C). At 5 dpf, in controls, 66% of the axonal length was myelinated, in comparison with 11% in TeNT-expressing neurons (Figure 2D). These data show that longitudinal growth of the myelin sheaths that did form was also reduced, indicating a role for vesicular release in the elongation of maturing myelin sheaths, complementing a recent study indicating a role for vesicular release in the very early elongation of nascent myelin sheaths [11]. In order to assess whether the effect on myelin sheath number

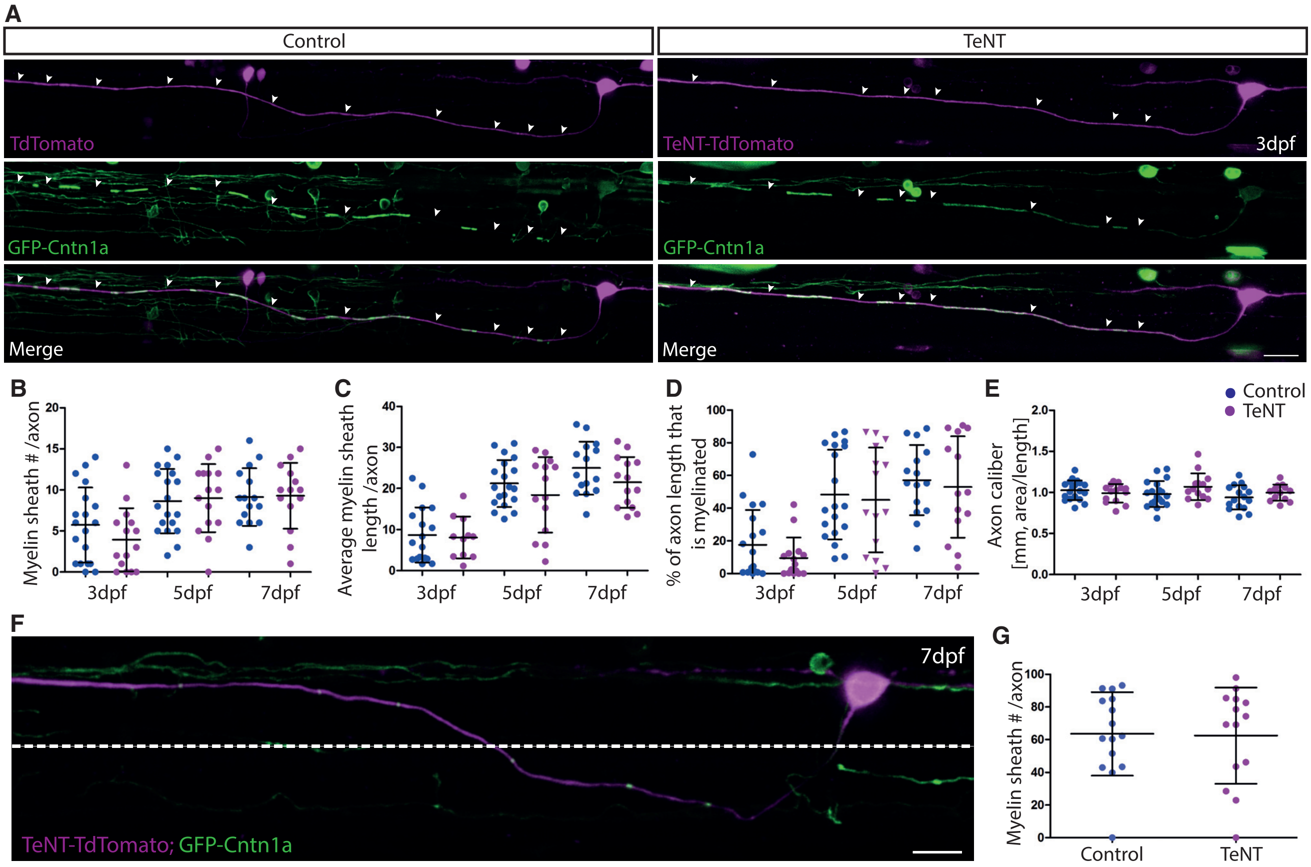

Figure 3. TeNT Expression in CoPA Neurons Does Not Impair Myelination along Individual Axons

(A) Individual CoPA axons labeled with GFP-cntn1a and TdTomato (left) and with GFP-Cntn1a and TeNT-TdTomato (right) at 3 dpf. Scale bar, 10 μm.

(B–E) Quantification of myelin sheath number per axon (B), average length of myelin sheath per axon per 425-μm imaging window (C), percentage of axon length (per 425-μm imaging window) that is myelinated (D), and axon caliber (E) at 3 dpf, 5 dpf, and 7 dpf in control and TeNT expressing CoPA neurons.

(F) Individual CoPA neuron and axon labeled with GFP-Cntn1a and TeNT-TdTomato. Dashed line indicates dorsoventral cutoff for axonal region analyzed when assessing region of CoPA axons in ventral spinal cord. Scale bar, 15 μm.

(G) Percentage of axon length that is myelinated in the ventral spinal cord of control and TeNT expressing CoPA neurons at 7 dpf. All error bars indicate ± SD. See also Figure S3.

and length might be a transient phenotype, we looked at myelination along the same axons at yet later stages. At 7 dpf, we found that myelination was still greatly reduced by disruption of vesicular release (sheath number: control 10.0 ± 2.5, n = 15 versus TeNT 6.2 ± 4.9, n = 14, p = 0.013; average sheath length: control 32.3 ± 9.2 μm versus TeNT, 13.9 ± 8.2 μm, $p < 0.0001$) (Figures 2A–2C). At this stage of the development of these individual neurons, an average of 77% of axonal length was myelinated in control versus just 23% in TeNT-expressing neurons (Figure 2D).

One possible explanation is that the reduction in myelination in TeNT-expressing reticulospinal neurons is due to an effect on axonal caliber, given that caliber itself has been implicated in regulating myelination [25–27]. We assessed axonal caliber along the length of the same individual GFP-Cntn1A, TdT expressing axons over time and found that control TdT- and TeNT-TdT-expressing neurons have similar axon calibers (3 dpf: control 1.1 ± 0.2 μm versus TeNT 1.1 ± 0.2 μm, p = 0.65; 5 dpf: control 1.2 ± 0.3 μm versus TeNT 1.2 ± 0.3 μm, p = 0.94; 7 dpf: control 1.1 ± 0.2 μm versus TeNT 1.3 ± 0.4μm, p = 0.31) (Figure 2E). In order to test for the possibility that subtle defects in caliber may be caused by disruption to vesicular release, we imaged axonal caliber at higher resolution using a new super-resolution confocal imaging modality (see the Experimental Procedures), which confirmed the similarity in caliber of TdT- and TeNT-TdT-expressing neurons (Figure S3).

Together, our data suggest that vesicular release directly regulates myelination along reticulospinal axons.

Expression of TeNT in CoPA Neurons Does Not Disrupt Myelination

We next tested whether vesicular release from CoPA neurons regulates myelination by expressing TeNT-TdT in these neurons as per reticulospinal neurons. In stark contrast, we found that expression of TeNT-TdT in CoPA neurons did not alter myelination (Figure 3). We found that myelin sheath number at 3 dpf, 5 dpf, and 7 dpf was similar along the axons of control and TeNT-expressing CoPA neurons (3 dpf: control 5.7 ± 4.6 sheaths, n = 19 versus TeNT 3.9 ± 3.9, n = 15, p = 0.23; 5 dpf: control 8.6 ± 4.0, n = 19 versus TeNT 9.0 ± 4.1, n = 15,

p = 0.79; 7 dpf: control 9.1 ± 3.5, n = 15 versus TeNT 9.3 ± 4.0, n = 14, p = 0.91) (Figures 3A and 3B). Similarly, we found that average myelin sheath length at 3 dpf, 5 dpf, and 7 dpf was also similar between control and CoPA axons (3 dpf: control 8.7 ± 6.7 μm versus TeNT 8.1 ± 5.1 μm, p = 0.80; 5 dpf: control 21.2 ± 5.7 μm versus TeNT 18.5 ± 9.2 μm, p = 0.29; 7 dpf: control 25.0 ± 6.4 μm versus TeNT 21.5 ± 6.2 μm, p = 0.15) (Figures 3A and 3C). Consequently, the proportion of the axonal stretches examined that were myelinated in control and TeNT-expressing CoPA neurons was similar at all stages examined (3 dpf: 18% versus 10%; 5 dpf 48% versus 45%; 7 dpf 57% versus 53%) (Figure 3D). As for reticulospinal axons, we did not find a difference in the caliber of CoPA axons between control and TeNT GFP-Cntn1A-expressing neurons (3 dpf: control caliber 1.0 ± 0.1 μm versus TeNT caliber 1.0 ± 0.1 μm, p = 0.38; 5 dpf: control caliber 1.0 ± 0.2 μm versus TeNT caliber 1.1 ± 0.2 μm, p = 0.12; 7 dpf: control caliber 0.9 ± 0.1 μm versus TeNT caliber 1.0 ± 0.1 μm, p = 0.23) (Figure 3E), even when imaged at super-resolution (Figure S3). These data indicate that CoPA neurons do not regulate their myelination via vesicular release in the same manner as reticulospinal neurons.

Differences in RS and CoPA Myelination Are Not Caused by Anatomical Positioning of Axons or Differential Function of TeNT

We wanted to investigate the possibility that the difference in myelination phenotypes between CoPA and reticulospinal could reflect the precise location of axons in the spinal cord. CoPA neuronal cell bodies are located in the dorsal spinal cord and project an axon to the ventral spinal cord that then crosses the embryonic midline before projecting dorsally within the dorsal longitudinal fasciculus (see Figures S1 and S2; Table S1). Thus, much of the proximal part of the CoPA axon resides in the ventral spinal cord in very close proximity to reticulospinal axons, which primarily project along ventrally located tracts of the spinal cord. Therefore, we assessed myelination along the stretches of CoPA axons that resided in the ventral spinal cord. Again, we found no difference in the average percent myelination between control and TeNT expressing neurons (7dpf: 64% control, versus 63% TeNT) (Figures 3F and 3G), indicating that the difference in myelination pattern does not relate to anatomical position and is specific to the individual neuronal cell type. To assess the relative ability of TeNT to disrupt synaptic vesicle release from reticulospinal and CoPA axons, we imaged vesicular release using sypHy [28]. sypHy is a fusion protein of the synaptic vesicle protein synaptophysin and the pH-sensitive variant of GFP, pHluorin [28]. sypHy allows assessment of synaptic vesicle release by virtue of the increase in pHluorin expression upon transition of the molecule from the acidic environment of the synaptic vesicle to the neutral environment of the extracellular space. We imaged non-evoked synaptic vesicle release events in the terminal collateral branches of individual TdT- and TeNT-TdT-expressing reticulospinal and CoPA neurons using sypHy (see the Experimental Procedures). We found that TeNT caused a significant reduction in the number of synaptic vesicle release events from the terminal axonal collateral branches of both reticulospinal and CoPA neurons, indicating that a differential function of tetanus between these neuronal subtypes does not underlie the distinct phenotypes that we observe on their myelination (Figure 4).

Our data show that distinct neuronal subtypes regulate myelination along the length of their axons in very different ways. Whereas TeNT-sensitive vesicular release profoundly regulates myelination of reticulospinal neurons, disruption of vesicular release does not affect myelination of CoPA axons. What accounts for the different modes of myelination of these two neuronal classes? The majority of reticulospinal neurons and CoPA neurons are glutamatergic [23], ruling out the simplest possibility that different major neurotransmitters are involved. Our analyses also revealed that both neuronal subtypes have similarly large caliber axons, ruling out axon caliber as the distinguishing characteristic. Also, both neuronal subtypes are born relatively early in the zebrafish CNS [20, 22], both extend long axons [17, 21], and reticulospinal and CoPA axons can even be myelinated by the same oligodendrocyte [19]. This begs the question as to whether neuronal subtype specific developmental programs and/ or functional properties determine whether a specific neuron regulates myelination in a vesicle-release-regulated manner or not. The recent documentation of distinct myelination patterns along the length of layer II–III cortical neurons, even when the radial position of these neurons is inverted in the cortex [13, 14], further suggests neuron-subtype-specific control of myelination. Furthermore, we have previously shown that supernumerary reticulospinal Mauthner neurons leads to an increase in myelin sheath number per oligodendrocyte [19], further implicating control of myelination by specific axons. In future studies, it would be interesting to perform gene expression profiling studies on different neuronal subtypes with distinct myelination patterns, e.g., reticulospinal and CoPA neurons, or different cortical layer neurons in mammals. It has also been shown that expression of axonal molecules implicated in myelination, such as neuregulin [8, 29], can be influenced by neuronal activity and even the animal's social environment [4, 30]. Therefore it would be informative to determine how the firing properties and mechanisms of vesicular release of distinct neurons, e.g., reticulospinal and CoPA neurons in zebrafish, differ at key developmental times and whether this relates to changes in the expression or localization of axonal cues and myelination. It will also be interesting to see how changes to myelination brought about by regulation of neuronal activity and vesicular release regulate the conduction properties of individual neurons by electrophysiological assessment at single-cell resolution.

Alternatively, the apparently different modes of myelination of distinct neuronal subtypes may be regulated through interactions with upstream or downstream neurons in their specific circuits. The number, spacing, length, and thickness of myelin sheaths along axons are all parameters that affect conduction time, and it is possible that such parameters are regulated along distinct axons for optimal circuit function rather than maximum velocity [31, 32]. But how could a neuron adapt its vesicular release to regulate myelin sheath growth so precisely as to achieve highly specific conduction times within a specific circuit? As a first step, the neuron or axon would need information that indicated that impulses were arriving either too slowly or too quickly for optimal circuit function in order to initiate a modulatory mechanism, events that would be compatible with spike-time-dependent plasticity [33]. Therefore, future functional

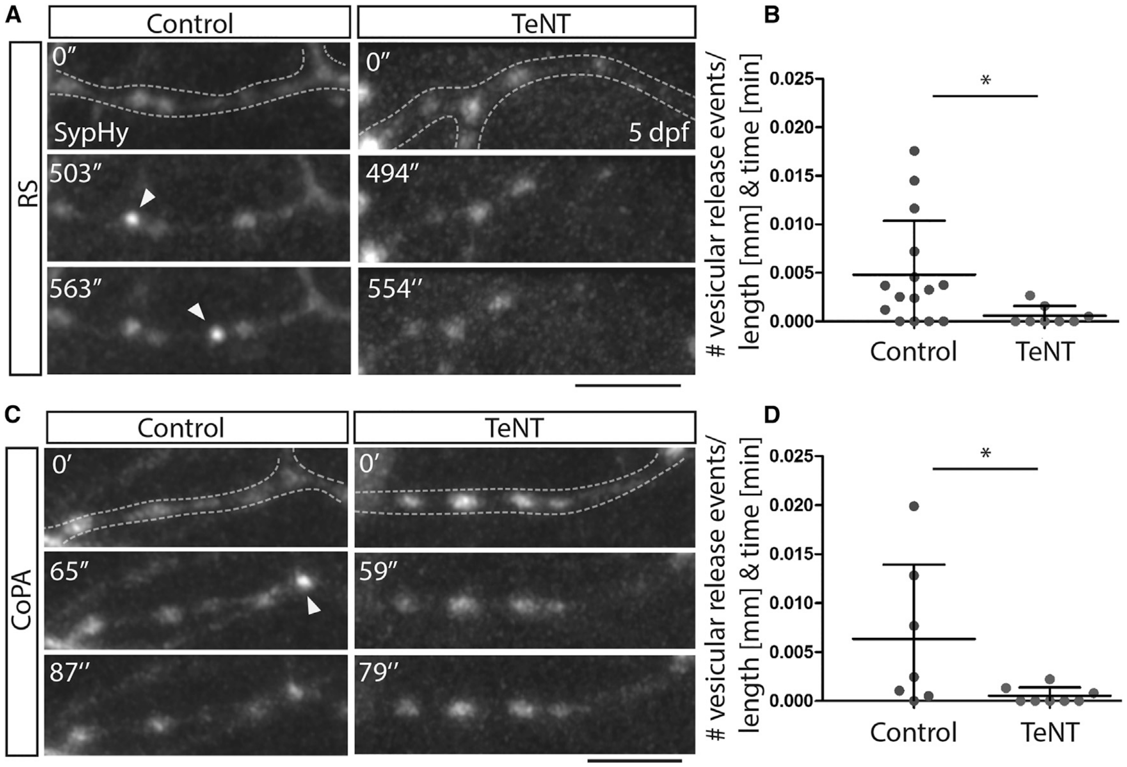

Figure 4. Tetanus Toxin Expression in Reticulospinal and CoPA Neurons Impairs Vesicular Release from Their Axons

(A) Images from time-lapse movies of sypHy expression in reticulospinal axon collaterals in control (left) and TeNT-expressing (right) neurons at 5 dpf. Dashed lines outline the collateral. Arrowheads point to punctate increases in GFP expression indicative of vesicular release. Scale bar, 5 μm.

(B) Quantitation indicates number of GFP events per collateral per micron per minute in control and TeNT-expressing reticulospinal neurons.

(C) Images from time-lapse movies of sypHy expression in CoPA axon collaterals in control (left) and TeNT-expressing (right) neurons at 5 dpf. Dashed lines outline the collateral. Arrowheads point to punctate increases in GFP expression indicative of vesicular release. Scale bar, 5 μm.

(D) Quantitation indicates number of GFP events per collateral per micron per minute in control and TeNT-expressing CoPA neurons.

See also Movies S1, S2, S3, and S4.

investigation of mechanisms of myelination may learn from investigations of synaptic plasticity and neuronal networks oscillations [34].

Recent evidence indicates that myelination continues well in to adult life and plays important roles in learning and memory (e.g., [3, 35]), but it remains unclear whether neuronal activity can play a direct role in myelin remodelling throughout life. Future studies employing conditional manipulation of firing or vesicular release in zebrafish could help provide novel insight into mechanisms of myelin plasticity by assessing how manipulation of activity or vesicular release from single axons that are already fully myelinated regulates myelin sheath maintenance and dynamic remodelling over time in the living animal. In the meantime, gaining a greater understanding of the molecular mechanisms of CNS myelination (both activity regulated and activity independent) is required before more sophisticated models of regulation can be proposed. For example, it is currently not clear which neurotransmitters or other possible vesicular release regulated signals or their cognate receptors regulate CNS myelination in vivo.

An alternative possibility is that regulation of myelination by synaptic vesicle release does not play a major role in fine-tuning the conduction properties of neuronal circuits, in which case one might consider alternative reasons for its existence. Vesicular release probability is regulated by numerous factors including action potential firing frequencies of individual neurons, and this may come at an energetic cost to the axon [36]. Consequently, it is possible that high-intensity firing or specific firing properties of distinct neurons that increase vesicular release may promote myelin sheath growth simply to provide additional metabolic support for the axon itself, a function that has recently been identified for myelin [37–39]. Therefore, it may be important to consider energy demand and axonal support when investigating the firing properties of individual neuronal subtypes and mechanisms of myelination.

In summary, our study indicates that distinct neurons can exhibit very different modes of vesicular release regulated myelination in vivo. This is of relevance to nervous system and neural circuit formation and function and will require extensive investigation to understand underlying mechanisms.

EXPERIMENTAL PROCEDURES

Zebrafish Lines

All animals were bred and maintained according to UK Home Office Guidelines. The transgenic lines Tg(sox10:mRFP) [40] and Tg(HuC:Gal4) [10] were

used in this project. The transgenic reporter Tg(mbp:mCherry-CAAX) was generated as part of this investigation.

Generation of Fluorescent Fusion Proteins to Visualize Myelination along Single Axons

Full-length *cntn1a* cDNA was amplified by RT-PCR from 3 dpf total RNA using primers 1 (EcoRI-Kozak-cntn1a-F, 5′-GAATTCCACCATGATTCCAGAGGCCTTCCAG-3′) and 2 (cntn1a-Stop-NotI-R, 5′-GCGGCCGCTCAGAGCATCAGAGTCCAGAG-3′); these primers added an EcoRI site followed by a Kozac consensus sequence (5′-CCACC-3′) to the 5′ end and a NotI site to the 3′ end. This fragment was cloned into pCRII-TOPO (Invitrogen) and subcloned into a modified version of pCS2(+). EGFP was inserted in the N-terminal region of Contactin1A, after amino acid position 45, to avoid interference with the GPI tether at the C terminus and ensure proper targeting. The first 135 bp encoding the cntn1a signal sequence were amplified with primers 1 (EcoRI-Kozak-cntn1a-F, 5′-GAATTCCACCATGATTCCAGAGGCCTTCCAG-3′) and 3 (cntn1a-sigpep-NcoI-R, 5′-CCATGGTCCCGAAGCCTGCAGACTC-3′). Second, 214 bp of *cntn1a* (encoding amino acids 46–116) were amplified using primers 4 (EGFP-BsrGI-cntn1a-F, 5′-GAGCTGTACAAGCCGGTGTTCGAGGAGCAG-3′) and 5 (cntn1a-seq-1R, 5′-CATGTTTGCTCTTGTCAG-3′). Primer 4 added the last 12 coding bps of EGFP. The N-terminal fusion was constructed through a 3-piece ligation of the *cntn1a* signal sequence region, EGFP (from pEGFP-N1, Clontech), and *cntn1a* sequence encoding amino acids 46–116. Sequencing confirmed that the reading frame is maintained throughout the fusion.

The GFP-Contactin1A fusion was subcloned into pBH-UAS, which contains two Tol2 sites that allow genomic insertion upon co-expression of tol2 transposase and 14 upstream activating sequences (UASs) to enhance expression.

Generation of Tg(mbp:mCherry-CAAX)

We have previously generated a p5E_mbp, which we have used to generate stable transgenic reporters of myelinating glia [19]. Here, we used the tol2kit [41] to recombine this regulatory sequence with the membrane-tethered red fluorescent protein mCherry-CAAX in pDestTol2CG2 using LR clonase II Plus (Invitrogen). Plasmid DNA (10 pg) was injected in to wild-type zebrafish eggs at the one-cell stage together with 25 pg mRNA encoding tol2 transposase to promote transgenesis. Founder animals were identified by screening F1 offspring. F1 offspring were raised to generate stable transgenic lines as in the past [19].

Labeling and Imaging Individual Neurons In Vivo

In order to label individual control and TeNT-expressing neurons in vivo, we injected single to 16-cell-stage embryos Tg(HuC:Gal4) embryos with 7 pg pBH-UAS-GFP-Contactin1A and 10 pg of either 5UAS-TdT or 5UAS-TeNT-TdT. For generation of the latter vectors, 5UAS repeats and a heat shock basal promoter were amplified from pCASPER and subcloned into pEGFP–N2 (Clontech) to generate p5UAS-EGFP–N2. TdT was subcloned into p5UAS EGFP–N2 to replace EGFP, thus generating p5UAS-TdT. For generation of a fusion of TeNT-Lc to TdT, the coding region for TeNT-Lc was subcloned into p5UAS-TdT at the N terminus of TdT. Plasmids were coinjected together with 25 pg mRNA encoding tol2 transpose to facilitate transgenesis [41]. Animals were mounted in 1.3% low-melting-point agarose prior to live imaging, released from agarose after imaging, kept individually, and remounted at 5 dpf and 7 dpf to generate time-course data. Specimens were imaged on Zeiss 710 and 780 laser scanning microscopes, using a 20× numerical aperture (NA) 0.8 lens. Fluorescent intensity profiles along the length of GFP-Cntn1a-expressing axons were plotted using ImageJ. Myelin sheaths were assessed by measuring gaps in GFP-Cntn1a localization along individual axons. We defined gaps in GFP-Cntn1a localization along the length of axons as myelin sheaths if these were complete gaps in fluorescence across the width of the axon. As well as representing the position and length of mature myelin sheaths, these gaps also include very short nascent myelin sheaths, some of which may be transient and retracted during dynamic stages of myelin sheath formation, as observed in previous studies [11].

Axon caliber was calculated from images taken at 20× by measuring three areas along each individual axon and dividing each of these three areas by its corresponding length. We then took an average of the three resultant diameters to estimate an average caliber for each individual axon. For super-resolution imaging of axonal caliber, we used a Zeiss LSM 880 Airyscan with a 63× NA 1.2 water-immersion lens, a post-magnification zoom of 1.8, and 3D deconvolution using a Wiener filter. To calculate caliber, we imaged two areas of each axon in super-resolution mode, divided each area by its length to assess diameter, and took an average of these diameters to estimate overall caliber.

Imaging Synaptic Vesicle Release Using sypHy

In order to image synaptic vesicle release in vivo, we used sypHy, a fusion of the synaptic vesicle protein synaptophysin and the pH-sensitive variant of GFP, pHluorin [28]. We subcloned sypHy into a pME vector compatible with the tol2kit and generated plasmids whereby sypHy was placed under the control of 10× UAS repeats.

We injected plasmid DNA (5 pg) encoding sypHy into Tg(HuC:Gal4) zebrafish embryos at the one-cell stage together with either UAS:TdT or UAS:TeNT-TdT. We screened for co-expression of TdT/ TeNT-TdT and sypHy in reticulospinal and CoPA neurons prior to imaging. Immediately prior to imaging sypHy, we treated animals with 0.3 mg/ml pancuronium bromide (P1918; Sigma) in embryo medium, in order to paralyze animals without affecting neuronal activity. We imaged sypHy on a Zeiss LSM 880 with Airyscan using a 20× lens NA 0.8 with a 1.8 post-magnification zoom. We screened animals for the presence of isolated terminal collateral branches in either reticulospinal or CoPA neuron axons. We used a fluorescence recovery after photobleaching (FRAP) protocol to image non-evoked vesicular release events using sypHy (three rounds of bleaching using the Zen2.1 Bleach function, interspersed by ten time-points of sypHy imaging). We acquired z stacks of 3–8 μm depth per time point, and imaged with time intervals from 7–22 s, depending on the frame size imaged. This relatively low temporal resolution precluded precise characterization of the kinetics of individual vesicular release events but allowed identification of synaptic vesicle release events. To analyze changes in fluorescence over time, we applied the ΔF/F formula $\Delta F/F = F(t) - F(0)/F(0) - Bg(t)$ to defined regions of interest, whereby F(t) refers to the fluorescence intensity at a specific time point (t), F(0) an average of the three time points following a bleach event, and Bg(t) the background fluorescence in a region of interest outwith the area of the collateral. We defined a vesicular release event as an increase in the ΔF/F value that was 5-fold greater than the SD in baseline fluorescence intensity in the region of interest. We normalized event number with respect to the length of collateral and duration of imaging. Each data point presented represents an average of two to four movies of one or two collaterals per axon.

Statistical Analyses

All data are expressed as mean ± SD. Statistical tests were carried out using GraphPad Prism5 or Microsoft Excel 2010. Two-tailed Student's t tests were applied throughout to assess significance. All analyses were performed by investigators blind to the experimental treatment.

SUPPLEMENTAL INFORMATION

Supplemental Information includes Supplemental Experimental Procedures, three figures, one table, and four movies and can be found with this article online at http://dx.doi.org/10.1016/j.cub.2016.03.070.

AUTHOR CONTRIBUTIONS

S.K. designed and performed experiments and co-wrote manuscript. M.G.V., R.G.A., M.B., J.S., and M.P.M. designed and validated transgenic constructs. W.S.T. supervised design of GFP-Cntn1a. D.A.L. designed experiments, analyzed data, managed the project, and co-wrote the manuscript.

ACKNOWLEDGMENTS

We would like to thank members of the D.A.L. lab, as well as Peter Brophy, Charles ffrench-Constant, Matthew Livesey, and Dies Meijer for helpful comments. We thank Leon Lagnado for sharing sypHy and Prof. Mike Cousin

and Dr. Karen Smillie for expert advice on imaging synaptic vesicle release. This work was supported by a principal's studentship to S.K., a NIH fellowship to M.G.V., an NIH grant (RO1 NS050223) to W.S.T., and a BBSRC David Phillips Fellowship, Lister Institute Research Prize, and Wellcome Trust Senior Research Fellowship to D.A.L.

Received: November 1, 2015
Revised: March 4, 2016
Accepted: March 31, 2016
Published: May 5, 2016

REFERENCES

1. Scholz, J., Klein, M.C., Behrens, T.E.J., and Johansen-Berg, H. (2009). Training induces changes in white-matter architecture. Nat. Neurosci. *12*, 1370–1371.
2. Zatorre, R.J., Fields, R.D., and Johansen-Berg, H. (2012). Plasticity in gray and white: neuroimaging changes in brain structure during learning. Nat. Neurosci. *15*, 528–536.
3. McKenzie, I.A., Ohayon, D., Li, H., de Faria, J.P., Emery, B., Tohyama, K., and Richardson, W.D. (2014). Motor skill learning requires active central myelination. Science *346*, 318–322.
4. Makinodan, M., Rosen, K.M., Ito, S., and Corfas, G. (2012). A critical period for social experience-dependent oligodendrocyte maturation and myelination. Science *337*, 1357–1360.
5. Demerens, C., Stankoff, B., Logak, M., Anglade, P., Allinquant, B., Couraud, F., Zalc, B., and Lubetzki, C. (1996). Induction of myelination in the central nervous system by electrical activity. Proc. Natl. Acad. Sci. USA *93*, 9887–9892.
6. Gibson, E.M., Purger, D., Mount, C.W., Goldstein, A.K., Lin, G.L., Wood, L.S., Inema, I., Miller, S.E., Bieri, G., Zuchero, J.B., et al. (2014). Neuronal activity promotes oligodendrogenesis and adaptive myelination in the mammalian brain. Science *344*, 1252304.
7. Wake, H., Lee, P.R., and Fields, R.D. (2011). Control of local protein synthesis and initial events in myelination by action potentials. Science *333*, 1647–1651.
8. Lundgaard, I., Luzhynskaya, A., Stockley, J.H., Wang, Z., Evans, K.A., Swire, M., Volbracht, K., Gautier, H.O.B., Franklin, R.J.M., Ffrench-Constant, C., Attwell, D., and Káradóttir, R.T. (2013). Neuregulin and BDNF induce a switch to NMDA receptor-dependent myelination by oligodendrocytes. PLoS Biol. *11*, e1001743.
9. Gautier, H.O.B., Evans, K.A., Volbracht, K., James, R., Sitnikov, S., Lundgaard, I., James, F., Lao-Peregrin, C., Reynolds, R., Franklin, R.J.M., and Káradóttir, R.T. (2015). Neuronal activity regulates remyelination via glutamate signalling to oligodendrocyte progenitors. Nat. Commun. *6*, 8518.
10. Mensch, S., Baraban, M., Almeida, R., Czopka, T., Ausborn, J., El Manira, A., and Lyons, D.A. (2015). Synaptic vesicle release regulates myelin sheath number of individual oligodendrocytes in vivo. Nat. Neurosci. *18*, 628–630.
11. Hines, J.H., Ravanelli, A.M., Schwindt, R., Scott, E.K., and Appel, B. (2015). Neuronal activity biases axon selection for myelination in vivo. Nat. Neurosci. *18*, 683–689.
12. Wake, H., Ortiz, F.C., Woo, D.H., Lee, P.R., Angulo, M.C., and Fields, R.D. (2015). Nonsynaptic junctions on myelinating glia promote preferential myelination of electrically active axons. Nat. Commun. *6*, 7844.
13. Tomassy, G.S., Berger, D.R., Chen, H.-H., Kasthuri, N., Hayworth, K.J., Vercelli, A., Seung, H.S., Lichtman, J.W., and Arlotta, P. (2014). Distinct profiles of myelin distribution along single axons of pyramidal neurons in the neocortex. Science *344*, 319–324.
14. Tomassy, G.S., Dershowitz, L.B., and Arlotta, P. (2016). Diversity matters: a revised guide to myelination. Trends Cell Biol. *26*, 135–147.
15. Rios, J.C., Melendez-Vasquez, C.V., Einheber, S., Lustig, M., Grumet, M., Hemperly, J., Peles, E., and Salzer, J.L. (2000). Contactin-associated protein (Caspr) and contactin form a complex that is targeted to the paranodal junctions during myelination. J. Neurosci. *20*, 8354–8364.
16. Çolakoğlu, G., Bergstrom-Tyrberg, U., Berglund, E.O., and Ranscht, B. (2014). Contactin-1 regulates myelination and nodal/paranodal domain organization in the central nervous system. Proc. Natl. Acad. Sci. USA *111*, E394–E403.
17. Kimmel, C.B., Powell, S.L., and Metcalfe, W.K. (1982). Brain neurons which project to the spinal cord in young larvae of the zebrafish. J. Comp. Neurol. *205*, 112–127.
18. Hale, M.E., Ritter, D.A., and Fetcho, J.R. (2001). A confocal study of spinal interneurons in living larval zebrafish. J. Comp. Neurol. *437*, 1–16.
19. Almeida, R.G., Czopka, T., Ffrench-Constant, C., and Lyons, D.A. (2011). Individual axons regulate the myelinating potential of single oligodendrocytes in vivo. Development *138*, 4443–4450.
20. Bernhardt, R.R., Chitnis, A.B., Lindamer, L., and Kuwada, J.Y. (1990). Identification of spinal neurons in the embryonic and larval zebrafish. J. Comp. Neurol. *302*, 603–616.
21. Easley-Neal, C., Fierro, J., Jr., Buchanan, J., and Washbourne, P. (2013). Late recruitment of synapsin to nascent synapses is regulated by Cdk5. Cell Rep. *3*, 1199–1212.
22. Mendelson, B. (1986). Development of reticulospinal neurons of the zebrafish. I. Time of origin. J. Comp. Neurol. *251*, 160–171.
23. Brustein, E., Saint-Amant, L., Buss, R.R., Chong, M., McDearmid, J.R., and Drapeau, P. (2003). Steps during the development of the zebrafish locomotor network. J. Physiol. Paris *97*, 77–86.
24. Remahl, S., and Hildebrand, C. (1990). Relations between axons and oligodendroglial cells during initial myelination. II. The individual axon. J. Neurocytol. *19*, 883–898.
25. Voyvodic, J.T. (1989). Target size regulates calibre and myelination of sympathetic axons. Nature *342*, 430–433.
26. Lee, S., Leach, M.K., Redmond, S.A., Chong, S.Y.C., Mellon, S.H., Tuck, S.J., Feng, Z.-Q., Corey, J.M., and Chan, J.R. (2012). A culture system to study oligodendrocyte myelination processes using engineered nanofibers. Nat. Methods *9*, 917–922.
27. Bechler, M.E., Byrne, L., and Ffrench-Constant, C. (2015). CNS myelin sheath lengths are an intrinsic property of oligodendrocytes. Curr. Biol. *25*, 2411–2416.
28. Granseth, B., Odermatt, B., Royle, S.J., and Lagnado, L. (2006). Clathrin-mediated endocytosis is the dominant mechanism of vesicle retrieval at hippocampal synapses. Neuron *51*, 773–786.
29. Brinkmann, B.G., Agarwal, A., Sereda, M.W., Garratt, A.N., Müller, T., Wende, H., Stassart, R.M., Nawaz, S., Humml, C., Velanac, V., et al. (2008). Neuregulin-1/ErbB signaling serves distinct functions in myelination of the peripheral and central nervous system. Neuron *59*, 581–595.
30. Liu, X., Bates, R., Yin, D.M., Shen, C., Wang, F., Su, N., Kirov, S.A., Luo, Y., Wang, J.Z., Xiong, W.C., and Mei, L. (2011). Specific regulation of NRG1 isoform expression by neuronal activity. J. Neurosci. *31*, 8491–8501.
31. Seidl, A.H. (2014). Regulation of conduction time along axons. Neuroscience *276*, 126–134.
32. Ford, M.C., Alexandrova, O., Cossell, L., Stange-Marten, A., Sinclair, J., Kopp-Scheinpflug, C., Pecka, M., Attwell, D., and Grothe, B. (2015). Tuning of Ranvier node and internode properties in myelinated axons to adjust action potential timing. Nat. Commun. *6*, 8073.
33. Feldman, D.E. (2012). The spike-timing dependence of plasticity. Neuron *75*, 556–571.
34. Fields, R.D., Araque, A., Johansen-Berg, H., Lim, S.-S., Lynch, G., Nave, K.-A., Nedergaard, M., Perez, R., Sejnowski, T., and Wake, H. (2014). Glial biology in learning and cognition. Neuroscientist *20*, 426–431.
35. Yeung, M.S.Y., Zdunek, S., Bergmann, O., Bernard, S., Salehpour, M., Alkass, K., Perl, S., Tisdale, J., Possnert, G., Brundin, L., et al. (2014). Dynamics of oligodendrocyte generation and myelination in the human brain. Cell *159*, 766–774.

36. Lewis, J.E., Gilmour, K.M., Moorhead, M.J., Perry, S.F., and Markham, M.R. (2014). Action potential energetics at the organismal level reveal a trade-off in efficiency at high firing rates. J. Neurosci. *34*, 197–201.

37. Fünfschilling, U., Supplie, L.M., Mahad, D., Boretius, S., Saab, A.S., Edgar, J., Brinkmann, B.G., Kassmann, C.M., Tzvetanova, I.D., Möbius, W., et al. (2012). Glycolytic oligodendrocytes maintain myelin and long-term axonal integrity. Nature *485*, 517–521.

38. Lee, Y., Morrison, B.M., Li, Y., Lengacher, S., Farah, M.H., Hoffman, P.N., Liu, Y., Tsingalia, A., Jin, L., Zhang, P.-W., et al. (2012). Oligodendroglia metabolically support axons and contribute to neurodegeneration. Nature *487*, 443–448.

39. Saab, A.S., Tzvetanova, I.D., and Nave, K.-A. (2013). The role of myelin and oligodendrocytes in axonal energy metabolism. Curr. Opin. Neurobiol. *23*, 1065–1072.

40. Kirby, B.B., Takada, N., Latimer, A.J., Shin, J., Carney, T.J., Kelsh, R.N., and Appel, B. (2006). In vivo time-lapse imaging shows dynamic oligodendrocyte progenitor behavior during zebrafish development. Nat. Neurosci. *9*, 1506–1511.

41. Kwan, K.M., Fujimoto, E., Grabher, C., Mangum, B.D., Hardy, M.E., Campbell, D.S., Parant, J.M., Yost, H.J., Kanki, J.P., and Chien, C.B. (2007). The Tol2kit: a multisite gateway-based construction kit for Tol2 transposon transgenesis constructs. Dev. Dyn. *236*, 3088–3099.

Current Biology, Volume *26*

Supplemental Information

Individual Neuronal Subtypes Exhibit

Diversity in CNS Myelination

Mediated by Synaptic Vesicle Release

Sigrid Koudelka, Matthew G. Voas, Rafael G. Almeida, Marion Baraban, Jan Soetaert, Martin P. Meyer, William S. Talbot, and David A. Lyons

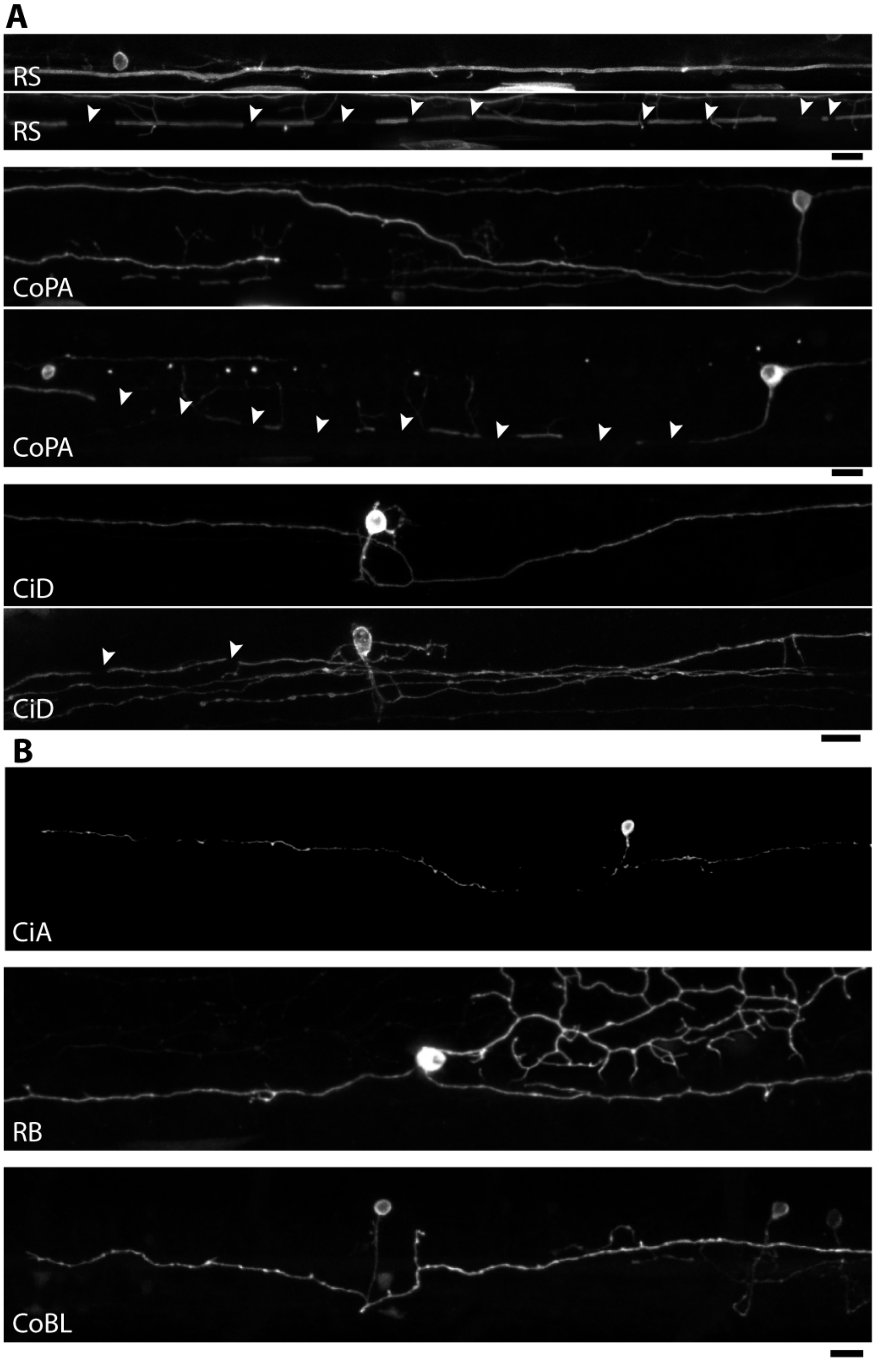

Figure S1 (relates to Figures 1, 2, 3).

Overview of individual subtypes labelled with GFP-Cntn1a that have myelinated axons (A) and that do not have myelinated axons (B) over the stages examined. A shows an example of each neuronal subtype at stages prior to myelination and upon myelination. Arrows point to position of myelin sheaths.

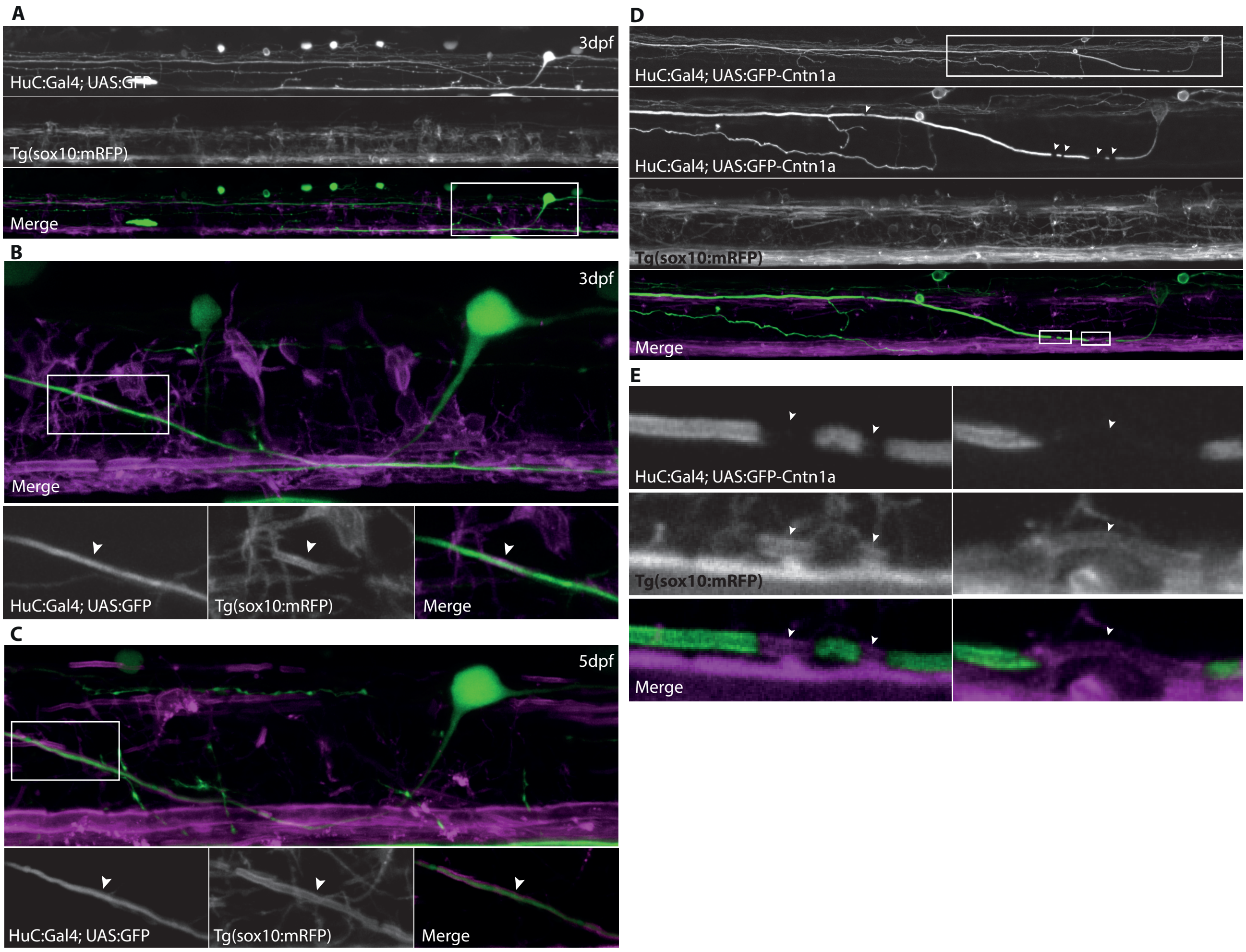

Figure S2 (relates to Figures 1 and 3).

Left panels: Individual CoPA neuron lablelled with cytoplasmic GFP in a Tg(sox10:mRFP) animal, imaged with a 10x magnification objective lens at 3dpf (A) and 63x (B) and also at 5dpf (C). Arrows indicate myelin sheaths. Area imaged in B outlined by inset in A.

Right panels: Individual CoPA neuron lablelled with GFP-Cntn1A in a Tg(sox10:mRFP) animal, imaged with a 10x magnification objective lens (D, top panel) 20x (D panels 2-4) and 63x (E). Arrows indicate myelin sheaths. Areas imaged in D outlined by insets in E.

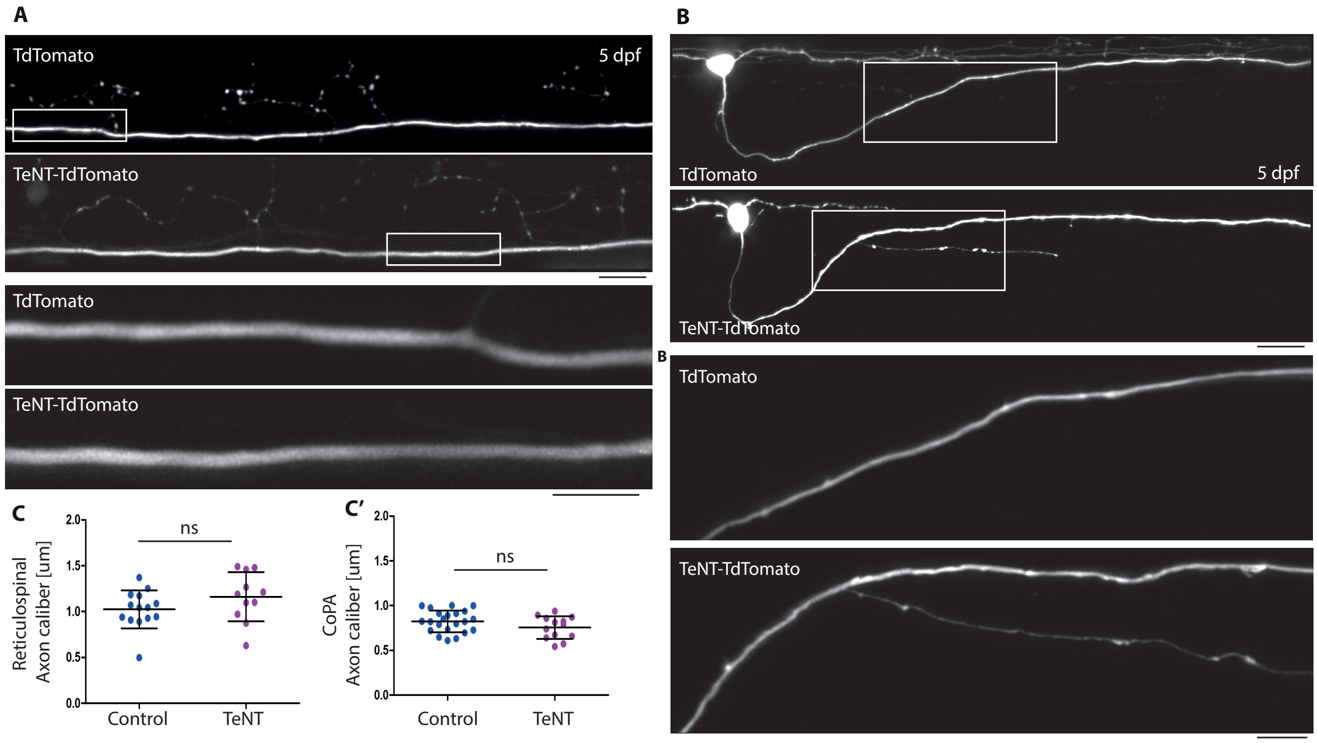

Figure S3 (relates to Figures 2, 3)

Individual reticulospinal axons (A) are of similar caliber when expressing control TdTomato (top) alone or TeNT-TdTomato (second from top) at 5 dpf. Scale bar: 15μm. Areas outlined by boxes in top and second panels are shown imaged by Airyscan in 3rd and 4th panels respectively. Scale bar: 5μm.

Individual CoPA axons (B) are of similar caliber when expressing control TdTomato (top) alone or TeNT-TdTomato (second from top) at 5 dpf. Scale bar: 20μm. Areas outlined by boxes in top and second panels are shown imaged by Airyscan in 3rd and 4th panels respectively. Scale bar: 5μm.

C and C' show quantification of axonal caliber and reveal no difference between control and TeNT expressing reticulospinal neurons (C) or CoPA (C').

Neuron cell type	Picture of neuron	Soma postition / # per hemi-segment	Maximum axon length	% of neurons with myelinated axons
Reticulospinal	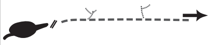	Ventral midbrain & hindbrain / axon projects through length of the spinal cord	Extends length of cord.	**Myelinated: 72% (28 out of 39)**
Commisural Primary Ascending (CoPA)	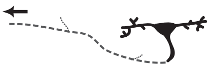	Dorsal, lateral to CoLA cell bodies / cell # varies in alternate segments	Extends length of cord / axon crosses cord dorsal to Mauthner axon. Ascend in dorsal cord.	**Myelinated: 82% (35 out of 43)**
Circumferental Descending (CiD)	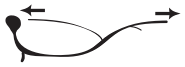	Midcord lateral / mutlitple cells per hemi-segment	13 segments/ axon ipsilateral, extends ventral to Mauthner axon then turns dorsal and caudally. Minor ascending branch.	**Myelinated: 53% (38 out of 72)**
Circumferental Ascending (CiA)	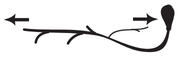	Midcord lateral / mutlitple cells per hemi-segment	Varies, the caudally projecting axon extends ipsilaterally over several segments. Minor descending branch.	Not myelinated: (0 out of 68)
Commisural Bifurcating Longitudinal (CoBL)	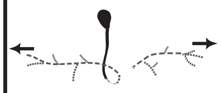	Dorsal / mutlitple cells per hemi-segment	4 segments/ axon projects ventrally, crossing cord dorsal to Mauthner axon before bifurcating and extending dorsally.	Not myelinated: (0 out of 40)
Rohon Beard (RB)	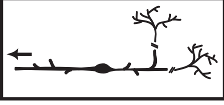	Dorsal of all other cells / 1-4 cells per hemi-segment	Caudally projecting axon extends > 20segments. Rostrally projecting axon extends 10 segments.	Normally not myelinated: 4% (2 out of 48, one short sheath each)

Table S1 (relates to Figures 1, 2, 3).

Summary of single neuron myelination profiling.

Table outlines neuronal subtypes whose myelination status at embryonic-early larval stages (up to 7 days post-fertilisation) was analysed, and provides schematic overview of characteristic morphology. The dashed lines indicate axons that cross the embryonic midline and double forward slashes indicate long distances between areas shown. Arrows indicate direction of axonal projection with anterior to the left and dorsal to the top.

Supplemental Experimental Procedures

In order to profile which neurons have myelinated axons during the first week of larval zebrafish development we mosaically labelled individual neurons with the GFP-Cntn1A transgenic reporter (Figure S1). We found that reticulospinal neurons, CoPA neurons and CiD (Circumferential Descending) neurons had myelinated axons during the first week of larval life (Figure S1+Table S1). To validate our results we also examined the myelination of individual neurons labelled by cytoplasmic GFP in a Tg(sox10:mRFP) background (Figure S2). Both analyses revealed prominent myelination of reticulospinal, CoPA and CiD neurons in the first week of life and no myelination of Circumferential Ascending, Commissural Bifurcating Longitudinal, and Rohon Beard axons (Table S1). Although these neurons did not have myelinated axons at the stages examined, we cannot rule out a later onset of myelination in these neurons. Furthermore, our analyses did not profile the myelination of every neuronal subtype with axons in the embryonic/ larval spinal cord, which will be important in future studies. Super-resolution imaging analyses of axonal caliber showed that control (TdTomato) and vesicular release impaired (TeNT-TdTomato) expressing neurons were of similar size (Figure S3).
